# Prediction of Glucose Concentration in Children with Type 1 Diabetes Using Neural Networks: An Edge Computing Application

**DOI:** 10.3390/bioengineering9050183

**Published:** 2022-04-21

**Authors:** Federico D’Antoni, Lorenzo Petrosino, Fabiola Sgarro, Antonio Pagano, Luca Vollero, Vincenzo Piemonte, Mario Merone

**Affiliations:** 1 Unit of Computer Systems and Bioinformatics, Department of Engineering, Università Campus Bio-Medico di Roma, 00128 Rome, Italy; lorenzo.petrosino@unicampus.it (L.P.); fabiola.sgarro1202@gmail.com (F.S.); paganoantonio9@gmail.com (A.P.); l.vollero@unicampus.it (L.V.); 2 Unit of Chemical Engineering, Department of Engineering, Università Campus Bio-Medico di Roma, 00128 Rome, Italy; v.piemonte@unicampus.it

**Keywords:** diabetes, time-series forecasting, glucose prediction, pediatrics, edge computing, neural network, decision support system, precision medicine, artificial intelligence

## Abstract

Background: Type 1 Diabetes Mellitus (T1D) is an autoimmune disease that can cause serious complications that can be avoided by preventing the glycemic levels from exceeding the physiological range. Straightforwardly, many data-driven models were developed to forecast future glycemic levels and to allow patients to avoid adverse events. Most models are tuned on data of adult patients, whereas the prediction of glycemic levels of pediatric patients has been rarely investigated, as they represent the most challenging T1D population. Methods: A Convolutional Neural Network (CNN) and a Long Short-Term Memory (LSTM) Recurrent Neural Network were optimized on glucose, insulin, and meal data of 10 virtual pediatric patients. The trained models were then implemented on two edge-computing boards to evaluate the feasibility of an edge system for glucose forecasting in terms of prediction accuracy and inference time. Results: The LSTM model achieved the best numeric and clinical accuracy when tested in the *.tflite* format, whereas the CNN achieved the best clinical accuracy in *uint8*. The inference time for each prediction was far under the limit represented by the sampling period. Conclusion: Both models effectively predict glucose in pediatric patients in terms of numerical and clinical accuracy. The edge implementation did not show a significant performance decrease, and the inference time was largely adequate for a real-time application.

## 1. Introduction

Type 1 Diabetes Mellitus (T1D) is a chronic disease in which the pancreas produces little or no insulin. If not treated properly, it can lead to both short- and long-term complications, including micro- and macro-vascular diseases that can damage kidneys, eyes, liver, and the circulatory system [1]. Although T1D has no cure, it can be managed through daily insulin administrations to keep the glycemic level in the euglycemic range, i.e., between 70 and 180 mg/dL. In recent years, the utilization of Continuous Glucose Monitoring (CGM) devices increased consistently because they allow patients to keep track of their glycemic trend 24 h a day.

The quality of life of people suffering from T1D improves considerably by preventing the blood glucose levels from exceeding the euglycemic range [2]. Albeit CGM devices have greatly enhanced the management of the disease [3], frequent hyperglycemic (CGM>180mg/dL), and hypoglycemic events (CGM<70mg/dL) are reported in clinical data. For this reason, in the last decade, many mathematical models have been developed to predict future glucose levels [4]. Indeed, an accurate forecast of the future glycemic level allows patients to adjust their therapy to prevent undesirable events. In particular, after having fixed a prediction horizon (PH), i.e., how forward in time the prediction is made, such models exploit the recent trends of CGM and other features such as the injected insulin to predict, through the medium of a regression task, what the glycemic level will be after PH minutes.

Although many physiological-based mathematical models exist to predict future glycemic levels, [5,6] the vast majority of recent research moved toward the implementation of data-driven models. In the latter case, whether machine learning and neural network/deep learning models have been implemented, the networks generally achieve better results [7]. In addition, some models capable of updating their training to catch more recent variations of the glycemic trend have been proposed [8]. The most widely used performance evaluation metric for blood glucose levels forecasting is the Root Mean Square Error (RMSE) which will be defined formally in Section 2.2. Briefly, the smaller the value, the better the performance.

In the frame of machine learning techniques, Bunescu et al. [9] use a three-compartmental physiological model of blood glucose dynamics to generate features for a Support Vector Regression (SVR) that is trained on patient-specific data. The model is validated on data of 5 T1D patients from a private dataset. The blood glucose levels forecasts with a 30- and 60-min PH attain RMSE values equal to 22.6 mg/dL and 35.8 mg/dL, respectively. Georga et al. [10] present a Random Forest regression technique for the personalized prediction of the glucose concentration in T1D patients. This multivariate model takes input CGM data, physiological features, and lifestyle information. High-accuracy forecasts are derived for a 15-min PH if all the available features are used (RMSE = 6.6 ± 1.3 mg/dL), whereas the performance considerably deteriorates when exploiting CGM data alone as input feature (RMSE = 11.3 ± 2.2 mg/dL). Sparacino et al. [11] propose a first-order AR model with time-varying parameters estimated at each timestamp using recursive least squares. They test several values of the forgetting factor with 30- and 45-min prediction horizons. The model is tuned on CGM data of 28 T1D patients from a private dataset. Results are accurate enough to potentially avoid or mitigate critical adverse events (RMSE = 18.3 ± 11.8 and 34.9 ± 21.3 mg/dL).

In the frame of neural networks and deep learning techniques, several well-established models have been applied to the task of glycemic prediction, achieving the best performance in the literature, and some brand new models have been proposed from scratch for this specific task [12,13]. Mosquera-Lopez et al. [14] present a Long Short-Term Memory (LSTM) recurrent neural network with a correction module to predict glycemic levels with a PH of 30 min, tuning the model on data of more than 4000 patients from a private dataset and testing it on data of further 10 patients, achieving an average RMSE 7.6 ± 2.2 mg/dL. Li et al. [15] propose a recurrent Convolutional Neural Network (CNN) to predict glycemic levels on simulated patients from the UVA/Padova simulator [16] and on 10 patients from private dataset with a PH of 30 and 60 min. They achieved better results for the simulated dataset (average RMSE = 9.4 ± 0.7 mg/dL and 18.9 ± 2.5 mg/dL) whereas performance degrades when testing on real data (RMSE = 21.1 ± 2.4 mg/dL for 30 min, 33.3 ± 4.8 mg/dL for 60 min).

Despite the large number of studies presented to forecast future glycemic levels and the noteworthy results they achieve, all the aforementioned papers focus on predicting the glycemic levels of adult subjects. Indeed, few works in the literature aim to predict blood glucose levels, specifically in pediatric patients. Children represent the most challenging diabetic population because pediatric patients go through a period of rapid growth, physiological and hormonal changes along with complex individualization and socialization processes. This often results in a significant decline in the quality of disease management, treatment adherence, and glycemic control [17,18]. Among the most remarkable studies, Mougiakakou et al. [19] test 2 different neural network models on real data of 4 T1D pediatric patients after pre-processing features with a glucose-insulin metabolism model. They achieve the best results (average RMSE = 22.2 ± 13.4 mg/dL) using a feedforward neural network. Dassau et al. [20] propose a hypoglycemia prediction algorithm that combines 5 different predictors to assess the risk of incoming hypoglycemia in the following 35 min in children with T1D, validating the system on 22 subjects. The decisions of the five models are combined through a majority vote, and the ensemble model identifies with sufficient advance 91% of the hypoglycemic events. Finally, De Bois et al. [21] test 6 different data-driven models on data of 10 virtual T1D children generated using the UVA/Padova simulator [16]. They generated for each patient 29 single days with a 3-meal daily scenario, exploiting the simulator’s built-in bolus calculator and treating each day as a standalone set of data. For a PH of 30 min, they achieve the best numerical performance using a Gaussian Process with a dot-product kernel (average RMSE = 5.2 ± 2.0 mg/dL). Conversely, the LSTM model results in the one with the greatest clinical accuracy, as 97.46% of its predictions fall into zones A and B of the Clarke Error Grid [22]) corresponding to accurate predictions.

Normally, machine learning techniques are validated in a laboratory setup, and when they are applied in practice, they are performed directly on servers or centralized processing units. The task of future glycemic levels prediction makes no exception, as most systems that perform real-time prediction exchange data between an edge device only used to gather information, and the cloud, where the actual glucose level forecasting is performed [23,24]. This is mainly due to the memory limits of edge-computing devices. Nonetheless, the drawback of such systems is that they constantly require an internet connection to work; this is not arguable with regards to medical devices because an interruption in the signal may result in missing decision support to the user. However, the increasing development of new, more powerful, and dedicated hardware, combined with the widespread use of IoT (Internet of Things) tools, enables the emergence of a branch of artificial intelligence known as inference at the edge [25,26]. This involves the machine learning models being run directly from a proximity device using data collected from associated sensors. Taking into account also the increasingly telemedicine-oriented approach [27,28], it is clear that the possibilities given by inference at the edge can be exploited to create predictive models that work in real-time with patient data to both improve the quality of life of patients and increase the ability of the physicians to extract useful information from the sensor data. Compared to systems that run on the cloud, edge computing can provide more reliable real-time service with low latency, and they are not limited by internet connectivity. For this reason, a recent study by Zhu et al. [29] proposed an Embedded Edge Evidential Neural Network to predict future glycemic levels of adult T1D patients in real-time exploiting CGM sensor readings and an edge-computing device. Due to limitations in the computational capacity, they converted their TensorFlow model to C and achieved an RMSE of 18.9 mg/dL with a PH of 30 min on both a public and a private dataset.

In the light of what is present in the literature, the contribution of this work is twofold. On the one hand, we implement two stat-of-the-art models for the prediction of glycemic levels and apply them to the specific task of the prediction in pediatric patients; such models improve the performance of the models currently studied in this field. On the other hand, we implement these models on an edge computing system, thus laying the foundations for the future creation of embedded devices capable of forecasting blood glucose levels to improve patients’ quality of life and aid medical diagnosis; we evaluate the feasibility of such prediction-at-the-edge system on two different boards in terms of prediction accuracy and execution time. To the best of our knowledge, this is the first attempt to implement a pediatric-specific glucose prediction model on an edge-computing system.

## 2. Materials and Methods

In this section, we present the generated dataset utilized to tune the predictive models, the description of the hardware that we used as an edge system for tests, and the experimental setup adopted with regard to the optimization of the neural network models as well as their implementation on the edge system.

### 2.1. Dataset

Data were produced for 10 pediatric patients by running several simulations in the UVA/Padova simulator [16]. Such a tool allows one to generate different scenarios for in silico patients by only providing a meal schedule. For each day of the simulation, we considered a baseline 5-meal schedule including 45, 20, 70, 20, and 80 g of carbohydrates ingested at times 8:00, 10:30, 13:00, 17:00, and 20:00, respectively. To make the simulation more realistic, each mealtime was shifted by an amount of time randomly chosen from a uniform distribution of ±60 min, whereas each amount of ingested carbohydrates was randomly modified by a number of grams taken from a uniform distribution of ±20 g. The simulator can determine the optimal insulin boluses to be injected for each meal of a specific patient and can thus provide the glycemic evolution for each subject for a pre-set number of days. However, the tool allows the user to modify the insulin bolus value and to include a sensor error in the CGM readings. Data are generated with a 1-min sampling.

Two different datasets were generated on a scenario consisting of 30 days of simulation with 5 meals per day. The first scenario has no errors in sensor reading and insulin administration, as automatically computed by the simulator, and thus corresponds to ideal T1D management. Differently, we created the second scenario using the same meal schedule as the first scenario, but by including CGM sensor errors and by forcing the presence of hyperglycemic and hypoglycemic events. We achieved such a goal by first allowing the UVA/Padova simulator to simulate with its own optimal bolus control; then, we extracted the vector of injected boluses and added random noise taken from a uniform distribution. In particular, each bolus consisting of *I* insulin units was modified according to the following:

(1) $$\hat{I}=I+z$$

where *z* is a random value taken in the interval [−3,3]. In practice, each bolus was increased or decreased by no more than 3 units of insulin from its optimal value. The modified bolus vector was given as an effective bolus vector to the UVA/Padova to run the simulations for this scenario. This makes such a scenario more realistic because, in real life, the increase or decrease in blood sugar levels occurs mainly due to an inaccurate estimate of the amount of carbohydrates ingested or to deviations in correction dosing [30]: we added noise on insulin boluses to simulate the human error.

**Figure 1:**
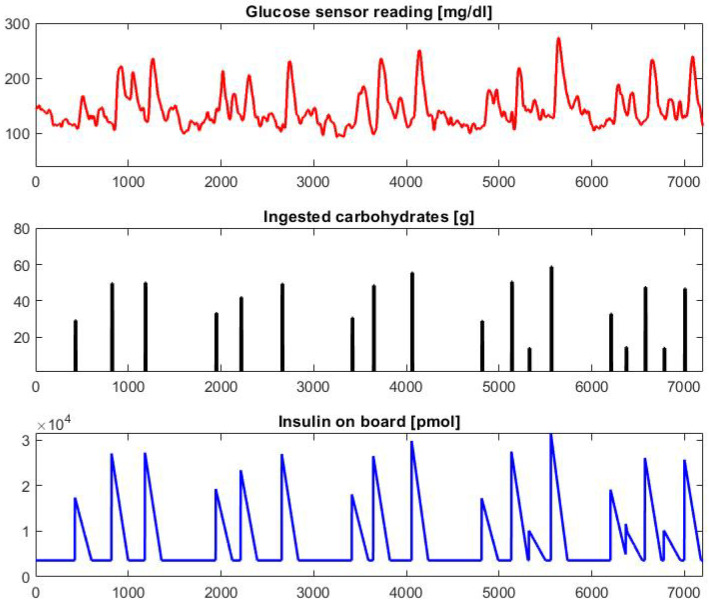
Graphical example of 5 days of data generated for patient child#007. Many hyperglycemic (G>180mg/dL) values can be observed due to the modification of the optimal bolus values.

The datasets consist of information on blood glucose levels and data on insulin (bolus, basal, and injection were added together and considered as a one) and finally, carbohydrate intake. Specifically, the final datasets consider Insulin-On-Board (*IOB*) as an insulin feature manually generated by exploiting a mathematical model [31]. *IOB* is a quantity that refers to the amount of rapid-acting insulin still active in the patient’s body after a bolus injection and thus provides deeper information on the recent history of insulin injections compared to the punctual insulin values themselves. The range of time for considering insulin still active is roughly between 2 and 8 h [32]. *IOB* is estimated differently among the main insulin pump companies, but in all cases, its calculation is based on insulin action plots which forecast the percentage of residual insulin as a function of time. For the Insulet pump, which is the one we selected when using the simulator, the active insulin time is equal to 3 h and the shape of the insulin action plot is linear [31]. Thus, the value of *IOB* for each timestamp *t* was computed as

(2) $$IOB\left(t\right)=\sum_{i=0}^{179}\alpha \left(i\right)u\left(t-i\right)$$

where u(t−i) represents the insulin injection at timestamp t−i, and α(i)=1−i/180 is the coefficient corresponding to the insulin decay curve. It is worth noting that only past insulin values (i.e., corresponding to timestamps ≤t) are used to compute the IOB. Specifically, 100% of the latest insulin injection value contributes to IOB(t), whereas the contribution linearly decreases to 0 for older values in the previous 3 h. Straightforwardly, the first 3 h of data of each patient were not used to train the predictive models, as they were used to initialize the *IOB* values. A graphical example of 5 days of data concerning the CGM sensor reading, the ingested carbohydrates, and the *IOB* of a sample patient generated with a 1-min sampling using the simulator and the pre-processing are reported in Figure 1.

### 2.2. Optimization of Network Models

A Precision Medicine approach was used to tune the predictive models, which involves choosing the hyper-parameters optimally and individually for each different subject. In this work, we implemented and optimized a CNN and an LSTM recurrent neural network because such models achieve the most promising performance in the literature [33]. Both networks were trained using a subset of the available data and then tested on subsequent data of the same in silico patient without being updated again. The networks have a sequence-to-label architecture, as the expected output is a single value corresponding to the expected blood glucose value in 30 min. After splitting the data into Training (70%), Validation (20%), and Test set (10%), the models were built.

**Figure 2:**
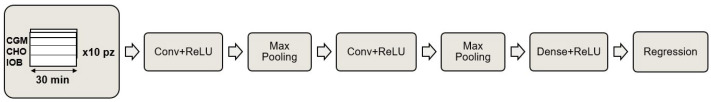
Schematic representation of the proposed Convolutional Neural Network.

The proposed CNN is a 1D-CNN with a one-dimensional kernel consisting of two convolutional layers with a ReLU activation function, each followed by a MaxPooling that cuts the parameters in half by taking, in pairs, only the largest value. To complete the model, the convolutional layers are followed by a dense layer with a ReLU activation function and an output neuron that provides the final regression. A schematic representation of the proposed CNN model is reported in Figure 2. The choice of hyper-parameters was made by performing a grid search on the validation set based on a range of parameters, including values identified through preliminary tests and parameters reported in the literature [33]. The optimization was done with respect to the kernel size and the number of feature maps.

**Figure 3:**
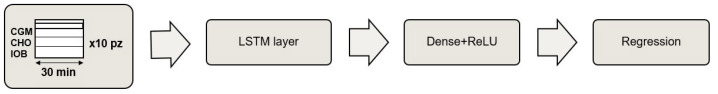
Schematic representation of the proposed LSTM Recurrent Neural Network.

The proposed LSTM model consists of a first LSTM layer, a dense layer with a ReLU activation function, and an output layer that returns the predicted CGM value. Moreover, in this case, the model was optimized in terms of the number of neurons in the first LSTM layer and the dense layer by investigating both parameters identified in preliminary tests and parameters reported in the literature [33]. A schematic representation of the proposed LSTM model is reported in Figure 3.

Both models take as input a (3×30) matrix of values, corresponding to the last 30 min of the 3 feature values. Such parameter was identified in preliminary tests, as it provides the models with enough information to capture the recent trend of the features. We found empirically that using longer monitoring periods did not improve performance. With regards to the strategy chosen to train both networks, the Stochastic Gradient Descent (SGD) optimizer is adopted, which requires a learning rate (0.0001), a momentum (0.9), and a clip Value (0.5), which is a necessary parameter to prevent the gradient explosion phenomenon in deep neural networks, improving the prediction quality. The training of both models was performed by splitting the data into mini-batches of 1400 samples (i.e., approximately one day of data) and setting the maximum number of epochs to 200. Finally, the early stopping strategy was adopted to prevent overfitting, which stops training if the performance on the validation set does not improve within a fixed number of consecutive epochs.

Two different evaluation metrics are used to thoroughly evaluate the performance of the models. Root Mean Square Error (RMSE)is utilized to assess numerical accuracy, as it provides a numerical estimate of how close the predicted values are to the real ones. Let us consider a prediction performed at timestamp *t*. Defined P(t + PH) as the prediction performed at time *t* regarding the future glucose value CGM(t + PH), and considering a time series with a total of *T* timestamps to be predicted, the RMSE is defined as:

(3) $$\mathrm{RMSE}=\sqrt{\sum_{t=1}^{T-PH}\frac{{\left(\mathrm{CGM}\left(t+PH\right)-P\left(t+PH\right)\right)}^{2}}{T-PH}}$$

where PH is the considered prediction horizon. The smaller the RMSE value, the better the performance. In addition, we considered the Clarke Error Grid (CEG) analysis as a measure of the clinical accuracy of the predictions produced. The CEG consists of a grid that is divided into 5 zones, from A to E, which plots the actual and the predicted CGM values on the horizontal and the vertical plot axis, respectively. Values in zones A and B represent good or acceptable glucose predictions; values in zone C represent mistaken predictions that may lead to unnecessary treatment; values in zone D represent a dangerous failure to predict; finally, values in zone E represent a completely wrong prediction that would lead to erroneous treatment [22].

### 2.3. Edge System Description

To test the feasibility of the predictive models of being implemented and utilized on an edge system, we needed to identify the target hardware. Our choice fell on two different devices: a Raspberry Pi4, chosen for its low cost and high computational capability, and a Coral DevBoard, a developer kit containing a Tensor Processing Unit (TPU) processor, which is useful for accelerating the execution of machine learning models. The Raspberry Pi4 has a Broadcom BCM2711 quad-core Arm Cortex A72 of 1.5 GHz processor, with 4 GB of memory. Furthermore, to carry out the tests, we used Raspbian OS (a Debian-derived ISO) as the operating system. Python and the Mendel Development Tool (MDT) were also installed. The former is necessary to perform tests directly on the Raspberry; the latter is used to give commands to the Coral DevBoard, which allows its set-up and use. The Coral Devboard has a quad Cortex-A53, Cortex-M4F CPU, with 1 GB LPDDR4 RAM, and it has a 4 TOPS (8 bit) TPU accelerator for machine learning processes. The operating system running on the DevBoard is Mendel Linux. We installed and utilized all the dependencies necessary to run the model on the board using the Py CoralAPI.

### 2.4. Edge System Implementation

Both datasets were provided as input, as sequences of the last 30 min of values, for two models compared: CNN and LSTM. The models were implemented and trained on Google Colab through the use of the open-source libraries of Keras and TensorFlow. Through this API, the networks were trained and the hyperparameters optimized.

Although the single models were trained on two different datasets, topologically the trained networks do not differ, in terms of hyperparameters. Therefore, the number of algebraic operations performed by a single network is invariant with respect to the dataset. Having made this consideration, we decided to implement on the edge device only the models trained on the dataset, including more hypo/hyperglycemic events, as it is more similar to a real use case.

For the implementation of the models on edge computing architectures, it is necessary to perform a quantization step that differs depending on the architecture on which inference is going to be performed. To perform regression tasks on the Rasperry, we used the quantization in *.tflite* format, which transforms the model keeping output variables in *float32* format. This optimization, namely dynamic range quantization, provides latency close to fully fixed-point inference. However, the outputs are still stored using floating-point so that the speedup with dynamic-range operations is less than a full fixed-point computation, as reported on the official TensorFlow web page [34]. From now on, we will refer to the model obtained with this quantization as *.tflite*.

For the implementation on the Dev Board, it was necessary to transform the models in their 8-bit representation to execute them, exploiting the full potential provided by Coral’s TPU. In this case, the quantization method is known as full integer quantization. Applying this approach requires one to provide a representative dataset to calibrate variable tensors such as model input, activation functions, outputs of intermediate layers, and model output. As a representative dataset, it would theoretically be sufficient to provide a set of 100–500 sample data taken between the training and validation set. In our case, a dependence of the goodness of the quantization on the subset of data passed to the model as a representative dataset was noted. In fact, it was not sufficient to use data taken randomly from the training or validation set but it was necessary to use ordered data, given the time series forecasting nature of the task. At the end of this quantization procedure, all input and output values are taken to *uint8*. From now on, we will refer to the model obtained with this quantization as *uint8*.

Due to the 8-bit nature of the quantization required to exploit the capabilities of the Coral Devboard TPU processor, a problem arose for our regression task. The range of values of the dataset varies between 10 and 600 mg/dL, whereas the values that can be represented with 8 bits are 256. Consequently, we pursued two approaches. The first consists of avoiding any pre-processing of the input data and then reconstructing the possible overflow cases obtained in the output through post-processing of the data, maintaining the granularity of the prediction at 1 mg/dL. The reconstruction was done following the procedure set out in the Algorithm 1. It assumes that a decrease of glucose concentration of more than 50 mg/dL in a single minute is very unlikely or impossible. In this case, we post-process the prediction and sum 255 to the predicted value.
**Algorithm 1** Output reconstruction algorithm1:reconstructed_pred = []                 ▹ initialization of variables2:overflow = False3:deltaY = 504:**For** i,x in enumerate (tflite_uint8_model_prediction):      ▹ Start of the for loop5:**if** x >= 240 **then**6:    **if** overflow and (x - tflite_uint8_model_prediction[i-1]) >= deltaY: **then**7:        overflow = False8:    **else if** not overflow and (x - tflite_uint8_model_prediction[i+1]) >= deltaY: **then**9:        overflow = True10:delta = 255 **if** overflow **else** 011:reconstructed_pred.append(x + delta)           ▹ End of the for loop

The second approach consists of the application of a normalization step in the pre-processing phase, remapping the data values between 0 and 255. Such an approach avoids problems related to overflow, but it takes the granularity of the prediction to approximately 2.33 mg/dL. Then, we de-normalized the predicted values to compute the evaluation metrics. This could introduce inaccuracy in the predictions.

**Figure 4:**
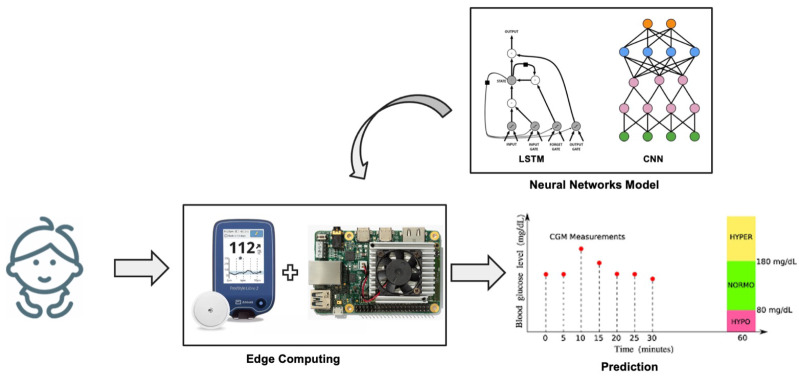
Schematic representation of the experimental setup during the test phase with edge systems.

The Raspberry and DevBoard were used for the calculation of inference times to be compared with the performance limits that our application requires (less than the sampling period of the sensor, i.e., 1 min). At each timestamp, the edge system takes as input the 30 most recent values of the features (i.e., the data of the in silico patient produced by the simulator), computes the latest value of the *IOB*, and performs a prediction of the future blood glucose level. A representative schematic of the experimental system can be seen in Figure 4.

## 3. Results and Discussion

As a result of the grid search performed on the Discovery set, the optimal configuration of the CNN comprises a number of filters equal to 26 for the first convolutional layer, 20 filters for the second convolutional layer, and a Kernel size equal to 1 × 5 on both. Note that, due to the shape chosen for the filters and the structure of the input matrix, in the first CNN layer, the convolutions are performed on different timestamps of the same feature. With regards to the LSTM model, the optimal configuration resulted in 64 neurons for both the LSTM and the fully-connected layer. Once the models were optimized, predictions were performed on the Test set, and the RMSE and the CEG were computed. With regards to the CEG values, only those from the second dataset were evaluated, as they present more hypo- and hyperglycemic values and are thus more similar to a real-life scenario.

Table 1 reports the average values and their standard deviation of the tests performed using the different versions of the models. As expected, the results achieved by the baseline model on the standard dataset are better than those achieved on the dataset with outliers. The LSTM model outperforms the CNN on both datasets, both in terms of average RMSE and CEG results. In particular, with regards to the realistic dataset, the LSTM achieves an RMSE of 16.3 ± 4.7 mg/dL, which is noteworthy if compared to other studies presented in the literature concerning the prediction of pediatric T1D patients. Also, 99.0% of its predictions fall in zones A and B of the CEG and thus represent clinically accurate or acceptable predictions, whereas 1.0% of predictions fall in zone D. The latter mainly correspond to failures in predicting hypoglycemia. No predictions fall in zones C and E.

A comparison with the results achieved in the literature can be only partial because, as explained in the Introduction, there are a limited number of studies addressing the prediction task on pediatric patients, and only one of them exploits the UVA/Padova simulator. The model tested on data from 4 real pediatric patients by Mougiakakou et al. [19] that achieves an average 22.1 mg/dL RMSE is outperformed by both the proposed models; however, it is known that forecasting glycemia of real patients is though compared to the virtual patient, because some unpredictable events might be present. De Bois et al. [21] tested the same 10 virtual children of the UVA/Padova simulator we utilized; they achieved an average RMSE of 5.2 mg/dL, which outperforms both the proposed models in all configurations in terms of numerical accuracy; nonetheless, the clinical accuracy of their best model (zones A + B) is 97.5% and it is outperformed by our models, which both achieve accuracy above 99.0% in their best configuration. However, it must be considered that the two datasets have been generated with a different meal and bolus schedules, so this comparison is just qualitative.

### Edge System Results and Discussions

**Figure 5:**
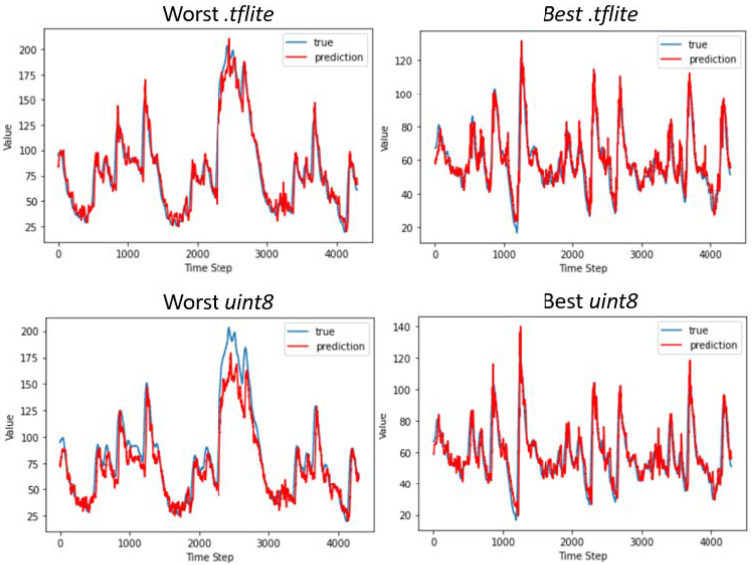
Graphical examples of the best and worst predictions performed by the CNN (**left**) and LSTM (**right**) using different edge devices. We computed the confidence interval for the predicted values, which are 2.01 for the worst *.tflite*, 2.14 for the worst *uint8*, and 1.09 for either the best *.tflite* and *uint8*, respectively. Nonetheless, we do not report such an interval in the figure because its values are too small to be observed in the graphics. The glycemic index values shown in the figure are normalized between 0 and 255; thus, to obtain the real glycemic values, we need to multiply by 2.33.

The results reported in Table 1 refer to the models trained without having carried out the normalization of the input values. The expected increase in the RMSE values of the models implemented on the edge devices can be observed; however, this variation differs between the two quantized representations of the networks. With regards to models quantized using dynamic range quantization for implementation on the Raspberry, the RMSE values increase by a maximum of 0.4 mg/dL for the CNN, whereas there is no difference for the LSTM. Again, the LSTM model outperforms the CNN in terms of numerical accuracy, achieving an RMSE of 16 ± 4.7 mg/dL, and 98.9% of its predictions fall in zones A and B of the CEG. This result is of particular interest because it is similar to the performance achieved on datasets composed of data of adult T1D patients, and it is achieved on the edge device without resorting to cloud computing. A graphical example of the predictions is reported in Figure 5, where we report as an example data of two patients for whom the best and the worst performance is achieved in terms of RMSE. The LSTM prediction is closer to the true CGM value compared to the CNN, which produces more oscillatory predictions; however, the LSTM tends to overestimate both hyperglycemic and hypoglycemic peaks.

**Figure 6:**
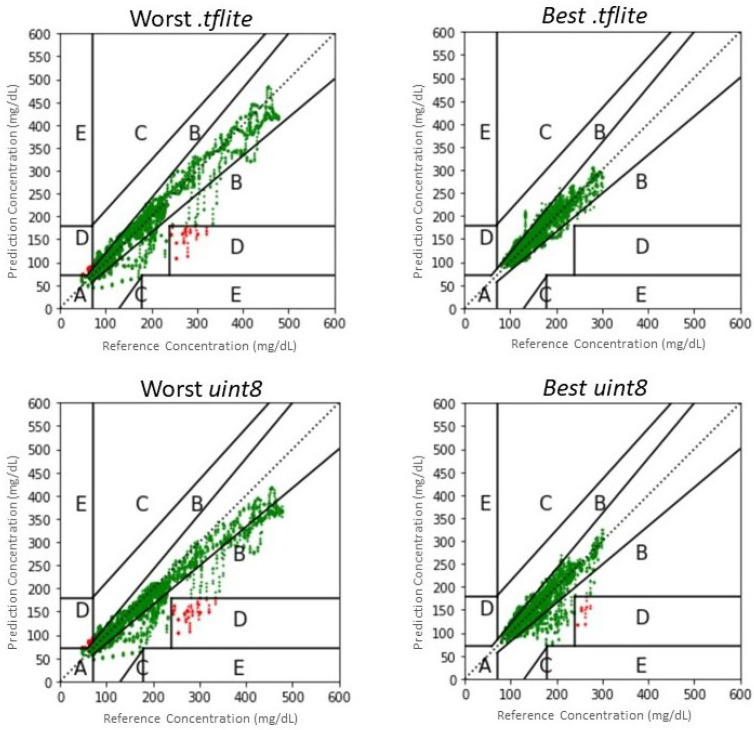
Clarke Error Grids resulted by the best and worst predictions of the CNN (**left**) and LSTM (**right**) using different edge devices. Predictions falling in the safe zones A and B are plotted in green; predictions in zone C are plotted in yellow; predictions falling in the dangerous zones D and E are plotted in red.

Nonetheless, it is worth noting that only 0.7% of predictions of the CNN model fall outside the A and B zones of the CEG, compared to 1.1% of the LSTM; conversely, the LSTM produces more predictions that fall in zone A (93.7% against 85.7% of the CNN). This may be explained considering that the LSTM is more capable of performing accurate predictions in the euglycemic range, which translates into better RMSE and a larger percentage of predictions in zone A, whereas it may miss some hypoglycemic events; on the contrary, the CNN has a larger RMSE and a larger amount of predictions in zone B of the CEG, corresponding to errors in the euglycemic range, whereas it is more capable of predicting hypoglycemia. Examples of the CEG are shown in Figure 6, where we report as an example data of two patients for whom the best and the worst performance is achieved in terms of CEG percentage in zone A. In conclusion, the CNN may be more appropriate to predict critical hypoglycemic events when implemented in *.tflite*, although its average numeric accuracy is worse than that of LSTM. However, it should be taken into account that results achieved on virtual patients are, in general, slightly better than those obtained on real patients; thus, performance may deteriorate when testing on a real dataset.

A different analysis applies to the models on which the full integer quantization was performed for implementation on the Coral DevBoard. Indeed, this quantization technique, which casts the values from *float32* to *uint8*, significantly affects the goodness of prediction. In particular, the overflow that is observed when glycemic values are above 255 mg/dL considerably increases the RMSE scores and generates some predictions that fall in the dangerous E zone of the CEG. For this reason, as explained in Section 2.4, two different approaches were chosen. The second one, which involved an initial pre-processing of the data, gave considerably better results than the first one, and they are reported in Table 2.

In particular, the results obtained for the models in Google Colab do not differ substantially from those achieved without the normalization; conversely, the *uint8* implementation of such models achieves considerably better performance than those obtained with the first approach. It must be considered that the granularity of the prediction increases from 1 mg/dL to 2.3 mg/dL. Despite this drawback, we can still consider this approach better than the first one because the increase in granularity obtained is not critical from a clinical point of view. It is worth noting that, although the LSTM model outperforms the CNN in terms of RMSE (21.2 ± 8.6 and 24.7 ± 5.5 mg/dL, respectively), 5% of the predictions produced by the LSTM fall in the D zone of the CEG, corresponding to a failure of predicting dangerous events. This situation shows that the LSTM model is weaker than the *uint8* representation, which brings it a greater drop in accuracy. This is probably due to the narrowness of the model, which has only one LSTM plane. Given the limited number of mathematical operations required to achieve an output, the conversion step of the model to *uint8* fails to optimize the weights with the new integer values. On the contrary, only 0.9% of the predictions produced by the CNN fall in the D zone, proving that this latter model is more clinically accurate and reliable when implementing the models in *uint8*, despite the better numerical accuracy achieved by the LSTM model.

A further comparison between the different implementations concerns the actual inference times obtained, which returned largely satisfying results. We reported in Table 3 the worst-case results for each model and hardware to show compliance with the time constraints posed by the application. The inference times for both models in all three representations are far below the limit imposed by the application, i.e., 1 min. However, the total times in the case of a real application should also consider the times necessary for: signal collection by the sensors, pre-processing of the raw data, and displaying the results on an appropriate Graphic User Interface (GUI). Nonetheless, the time for a single inference operation to be summed is, in the worst case, the ones of the CNN performed in the *.tflite* format by the Raspberry, corresponding to 101.56 ms. We can therefore assert that inference times, covering at most 0.17% of the total time limit imposed by the application, are not one of the parameters to be optimized in the case of a real implementation of the system. Furthermore, looking at Table 3 and comparing the data obtained in the tests of the two Edge systems, a consistent acceleration can be observed with the use of the Coral DevBoard when compared to the Raspberry’s performance, although it does not reach the performance of Google Colab TPU. This result is in line with Google’s own claims [35].

## 4. Conclusions

In this manuscript, we implemented a CNN and an LSTM neural network for the prediction of blood glucose concentration in pediatric T1D patients. The UVA/Padova simulator was exploited to generate data of 10 virtual children, and 2 datasets were generated which differ in the amount of hypoglycemic and hyperglycemic events. We determined the optimal parameters of the models through the medium of a grid search on the Discovery set and evaluated performance by the predictions on the Test set using Google Colab, a Raspberry, and a Coral DevBoard. To the best of our knowledge, this is the first attempt to implement an edge-computing system for the prediction of glucose concentration in children.

With regards to the prediction of glucose levels, the models achieved numerical accuracy comparable to those reported in the literature for adult patients. However, we acknowledge that, since the results are achieved on virtual patients, they may not be fully representative of the actual predictive capabilities of the models. On the one hand, the LSTM model achieved the best numerical accuracy and the largest percentage of predictions in zone A of the CEG for all the tests performed without model quantization. On the other hand, the CNN model produced a smaller percentage of predictions in the dangerous zones of the CEG with respect to all the implementations on edge devices, proving to be more effective in predicting critical events. In conclusion, both proposed models are promising for possible real implementation in pediatric patients.

With regards to edge computing, we arrived at a double result. On the one hand, the loss of information and prediction quality was tested with respect to two different quantizations of the networks. Both approaches achieved results comparable to those achieved using Google Colab. The *.tflite* implementation achieved the best results, although the *uint8* showed smaller inference times. On the other hand, the tests on inference times showed us that the IoT devices currently on the market have sufficient computational capabilities to be used in applications that require time constraints such as the one imposed by our specific case study, i.e., 1 min. In conclusion, the *.tflite* implementation seems more promising because it achieves the best results and there is no particular concern about the inference time.

Several future developments may follow this work. First, it would be interesting to validate the proposed neural networks on data of real patients to confirm the good performance achieved on virtual patients. Second, a mobile application could be developed to provide the patient with real-time information about their future glycemic value and generate an alarm in case of dangerous conditions by directly interacting with the edge device. Such application may also collect a history of the patient’s data to allow the physicians to adjust the therapy. Finally, it would be interesting to develop a complete proof of concept, including also the acquisition system, to exploit its actual limits and potential.

## Figures and Tables

**Table 1:** Results of the tests performed with the proposed models CNN and LSTM. In this test, the normalization step was not performed in the pre-processing phase. The results refer to the RMSE [mg/dL] achieved on both the ideal (no-error) and the realistic (hypo-hyper) dataset. Such results are reported in terms of average RMSE ± standard deviation. The CEG results are referred only to the realistic dataset, and its results are reported as percentage on the total dataset. For each neural network, we reported the results for the model implemented on Google Colab, for the model implemented on Raspberry (*.tflite float32* format), and for the model implemented on the Dev Board (*.tflite uint8*).

Model	RMSE (No-Error)	RMSE (Hypo-Hyper)	CEG (A; B; C; D; E)
CNN	22.2±2.5	23.2±2.3	87.0; 12.0; 0.0; 1.0; 0.0
LSTM	13.5±3.4	16.3±4.7	93.8; 5.2; 0.0; 1.0; 0.0
CNN *.tflite*	/	23.6±2.0	85.7; 13.6; 0.0; 0.7; 0.0
LSTM *.tflite*	/	16.3±4.7	93.7; 5.2; 0.0; 1.1; 0.0
CNN *uint8*	/	40.1±11.1	75.4; 20.8; 0.0; 1.2; 2.5
LSTM *uint8*	/	35.0±13.3	82.4; 12.5; 0.0; 1.5; 3.6

**Table 2:** Results of the tests performed with the proposed models CNN and LSTM, on which was carried the normalization step in the pre-processing phase. The results refer to the RMSE [mg/dL] achieved on the realistic (hypo-hyper) dataset. Such results are reported in terms of average RMSE ± standard deviation. The CEG results are referred only to the realistic dataset, and its results are reported as percentage on the total dataset. For each neural network, we reported the results for the model implemented on Google Colab, and for the model implemented on the Dev Board (*.tflite uint8* format).

Model	RMSE (Hypo-Hyper)	CEG (A; B; C; D; E)
CNN	21.8±2.3	87.8; 10.9; 0.0; 1.1; 0.0
LSTM	16.0±3.4	93.7; 5.5; 0.0; 0.8; 0.0
CNN *uint8*-normalized	24.7±5.5	87.6; 9.8; 0.0; 0.9; 0.0
LSTM *uint8*-normalized	21.2±8.6	87.4; 7.5; 0.0; 5.1; 0.0

**Table 3:** Maximum inference time obtained in the test phase in milliseconds. The inference times are reported for each model, CNN and LSTM. They were calculated: for the models saved in TensorFlow saved model format over the Colab online TPU, for the *.tflite* model format over the Raspberry and for the *.tflite* format quantizated in *uint8* over the Coral DevBoard.

Model	Colab TPU (TF Saved Model)	Raspberry (*.tflite*)	Coral DevBoard (*.tflite* *uint8*)
CNN	0.085	101.56	18
LSTM	0.086	70.3	12

## Data Availability

Restrictions apply to the availability of these data. Data was obtained from The Epsilon Group and are available at https://tegvirginia.com/software/t1dms-2014/ (accessed on 20 November 2021) with the permission of The Epsilon Group.

## Sample Availability

Samples of the scenario generation codes are available from the authors.
